# Associations of Fat Mass and Fat-Free Mass with Physical Fitness in 4-Year-Old Children: Results from the MINISTOP Trial

**DOI:** 10.3390/nu8080473

**Published:** 2016-07-30

**Authors:** Pontus Henriksson, Cristina Cadenas-Sanchez, Marja H. Leppänen, Christine Delisle Nyström, Francisco B. Ortega, Jeremy Pomeroy, Jonatan R. Ruiz, Marie Löf

**Affiliations:** 1 PROmoting FITness and Health through Physical Activity Research Group (PROFITH), Department of Physical Education and Sport, Faculty of Sport Sciences, University of Granada, 18071 Granada, Spain; cristina.cadenas.sanchez@gmail.com (C.C.-S.); ortegaf@ugr.es (F.B.O.); ruizj@ugr.es (J.R.R.); 2 Department of Clinical and Experimental Medicine, Faculty of Health Science, Linköping University, 581 53 Linköping, Sweden; 3 Department of Health Sciences, University of Jyvaskyla, 40014 Jyvaskyla, Finland; marja.h.leppanen@gmail.com; 4 Department of Biosciences and Nutrition, Karolinska Institutet, 141 83 Huddinge, Sweden; christine.delisle.nystrom@ki.se; 5 Marshfield Clinic Research Foundation, Marshfield, WI 54449, USA; pomeroy.jeremy@mcrf.mfldclin.edu

**Keywords:** air-displacement plethysmography, body composition, cardiorespiratory fitness, muscular strength, motor fitness, preschool

## Abstract

Physical fitness is a powerful marker of health in youth. Studies in adolescents and adults suggest that higher fat mass is related to worse physical fitness. However, there is limited knowledge whether fat mass and fat-free mass are associated with physical fitness already in preschoolers. Baseline data from the MINISTOP (Mobile-based INtervention Intended to STop Obesity in Preschoolers) trial was utilized for this cross-sectional analysis. Body composition was assessed using air-displacement plethysmography. Fat mass index [fat mass (kg)/height^2^ (m)] and fat-free mass index [fat-free mass (kg)/height^2^ (m)] were used to provide height-adjusted measures of body composition. Physical fitness was measured using the PREFIT (FITness testing in PREschool children) battery, which assesses cardiorespiratory fitness, upper-body and lower-body muscular strength as well as motor fitness. In total, this study included 303 children (168 boys and 135 girls), who were on average 4.48 ± 0.15 years old. Higher fat mass index was associated with worse cardiorespiratory fitness (standardized β = −0.17, *p* = 0.002), lower-body muscular strength (β = −0.17, *p* = 0.003) and motor fitness (β = −0.21, *p* < 0.001) in regression analyses adjusted for age, sex and mutually adjusted for fat-mass index and fat-free mass index. Conversely, higher fat-free mass index was associated with better cardiorespiratory fitness (β = 0.18, *p* = 0.002), upper-body muscular strength (β = 0.39, *p* < 0.001), lower-body muscular strength (β = 0.22, *p* < 0.001) and motor fitness (β = 0.17, *p* = 0.004). Thus, fat mass and fat-free mass in preschoolers appear to have joint but opposite associations with physical fitness, an important marker for current and future health.

## 1. Introduction

Childhood obesity is a serious public health challenge [1] and the proportion of overweight and obese children is high in many countries [1,2]. This is of great concern since childhood obesity is associated with a higher risk of adult obesity [1,3] as well as impaired health [4] later in life. A high body fatness in childhood may also have a negative impact on physical fitness [5,6,7,8], which is a potentially important observation since physical fitness is a powerful marker of health already in youth [4,9,10]. For instance, higher levels of physical fitness (particularly cardiorespiratory fitness and muscular strength) in childhood and adolescence have been associated with a healthier cardiovascular profile later in life and with a lower risk of premature death [4,9,10]. Although there is some evidence that body fatness may be related to physical fitness in primary school children [5,7,8], very little is known whether such associations are present already in preschool children, i.e., children aged 5 years or younger.

The few studies conducted in preschoolers suggest that a higher body mass index (BMI) is associated with a lower cardiorespiratory fitness [11,12,13,14,15] but with a higher upper-body muscular strength [12,16]. Further, conflicting results have been presented whether BMI is associated with lower-body muscular strength and measures of motor fitness/speed-agility [12,13,15,16,17]. However, BMI is poorly correlated with % fat mass (%FM) in a preschool population [18]. Furthermore, BMI reflects both the fat mass (FM) and the fat-free mass (FFM) in the body [19], which may have different associations with physical fitness. To our knowledge, only two studies [14,15] have reported any data on the association between estimates of FM with physical fitness in preschoolers. Furthermore, we have not found any studies that have investigated associations between FFM and physical fitness in preschoolers. Consequently, further studies investigating associations of both FM and FFM with physical fitness are warranted in preschoolers. The aim of this study was therefore to investigate the associations of FM and FFM with physical fitness in preschool-aged children.

## 2. Materials and Methods

### 2.1. Design and Participants

This study was performed under the umbrella of the MINISTOP project (Mobile-based INtervention Intended to STop Obesity in Preschoolers) [20,21]. Briefly, the MINISTOP project is a randomized controlled trial conducted in Östergötland (Sweden) between 2014 and 2015. The objective was to evaluate the impact of a mobile-based parental health intervention on body fatness, dietary habits, physical fitness, physical activity and sedentary behaviour in 4-year-old children [20]. This analysis only includes baseline data collected prior to randomization. Of the 315 preschoolers assessed at baseline, 303 had complete body composition data and were included in this study. Parents reported their age, weight, height and educational attainment using a questionnaire. The study was conducted according to the guidelines laid down in the Declaration of Helsinki and informed consent, witnessed and formally recorded, was obtained from all parents. The MINISTOP trial was approved by the Research Ethics Committee, Stockholm, Sweden (2013/1607–31/5; 2013/2250–32) and is registered as a clinical trial (https://clinicaltrials.gov/ct2/show/NCT02021786).

### 2.2. Body Composition

Weight was measured to the nearest gram using an electronic scale attached to the BodPod (COSMED USA, Concord, CA, USA). Height was measured using a wall stadiometer (Tillquist, Spånga, Sweden) to the nearest 0.1 cm. Overweight/obesity was classified according to Cole et al. [22]. Weight-for-age and length-for-age z-scores were calculated using Swedish reference data [23]. FM and FFM were assessed using air-displacement plethysmography using the pediatric option for BodPod (COSMED USA, Concord, CA, USA), which has been shown to be accurate in the estimation of %FM in preschool children [24]. The complete test-procedure has been described elsewhere [18,24]. All measurements were performed without shoes and in tight fitting underwear. BMI was calculated as body weight (kg)/height^2^ (m). Fat mass index (FMI) [FM (kg)/height^2^ (m)] and fat-free mass index (FFMI) [fat-free mass (kg)/height^2^ (m)] were calculated to provide height-adjusted measures of FM and FFM, respectively.

### 2.3. Physical Fitness

Cardiorespiratory fitness, muscular strength and motor fitness were assessed by the PREFIT (FITness testing in PREschool children) battery [25]. Detailed information of each test has been reported elsewhere [26]. Cardiorespiratory fitness was measured using the 20 m shuttle run test which started at 8.5 km/h and increased 0.5 km/h/min. Each child ran the 20 m shuttle run test individually and one person from the trained research staff ran next to the child in order to pace them. Upper-body muscular strength was assessed by the handgrip strength test using an analogue dynamometer (TKK 5001, Grip-A, Takei, Tokyo, Japan) with a grip span of 4.5 cm. For lower-body muscular strength, the standing long jump test was used. Motor fitness and speed-agility was assessed by the 4 × 10 m shuttle run test. All fitness tests were conducted twice, apart from the 20 m shuttle run test that was performed once. In the handgrip strength test, the best of two attempts for each hand was selected, and the average of both hands was calculated and used in the analyses. For the standing long jump and the 4 × 10 m shuttle run tests, the best of two attempts was used.

### 2.4. Physical Activity

As described previously [21], the children wore the ActiGraph wGT3x-BT triaxial accelerometer (www.actigraphcorp.com) on the non-dominant wrist for 24 h during seven consecutive days. A valid day was ≥600 min of awake wearing time [27] and children with ≥3 days of valid data were used in the analyses. Time in vigorous-intensity physical activity (VPA) was calculated from the sum of vector magnitudes using a cut-point of ≥1969 vector magnitude per 5 s [28].

### 2.5. Statistical Analysis

To investigate associations between body composition (x) and physical fitness (y), linear regression analyses were conducted. Three regression models were created: (1) unadjusted model; (2) model adjusted for age and sex of the child, and models with FMI and FFMI as independent variables were also mutually adjusted for FMI and FFMI; and (3) model adjusted for the potential confounders described in model 2 plus the amount of time spent in VPA (min/day) since VPA has previously been associated with physical fitness in preschoolers [21,29]. Further adjustments of all regression models for maternal and paternal variables (age, BMI, educational attainment) had very little influence on the estimates. We further examined sex-interactions between body composition and sex by including interaction terms (i.e., body composition measure × sex) separately in the regression models. *p* was > 0.05 for all interaction terms and consequently, we present the results for boys and girls together. Independent *t*-tests or chi square tests were applied to test differences between groups. All hypothesis tests were two-sided and a *p* < 0.05 was considered statistically significant. Statistical analysis was performed using SPSS Statistics 22 (IBM, Armonk, NY, USA).

## 3. Results

### 3.1. Descriptive Statistics

Mothers of the children in the study were on average 36 ± 4 (mean ± SD) years old, had an average BMI of 24.1 ± 4.8 kg/m^2^, and 71% of mothers had a university degree. Fathers were on average 38 ± 5 years old, had an average BMI of 25.2 ± 4.3 kg/m^2^, and 58% had a university degree. Table 1 presents the age, anthropometric variables, body composition and physical fitness of the 303 children.

### 3.2. Associations of Body Composition with Physical Fitness

Table 2 reports the associations between body composition (x) and physical fitness (y). BMI was positively associated with handgrip strength (*p* < 0.001) but had no statistically significant associations with any other physical fitness variables. In adjusted analysis, each unit (kg/m^2^) increase in BMI was associated with a 0.38 kg (*p* < 0.001) increase in the handgrip strength test. Further adjustments for VPA had little influence on the associations of BMI with physical fitness. Using body weight instead of BMI yielded very similar results in the analyses. Thus, body weight (kg) was associated with handgrip strength (kg) in the adjusted model (b = 0.32, standardized β = 0.50, *p* < 0.001) but not with any other physical fitness measures (*p* > 0.05). Finally, results were also comparable when comparing non-overweight and overweight children (Table S1). Thus, overweight children had a higher average handgrip strength as compared to non-overweight children (7.5 kg vs. 6.3 kg, *p* < 0.001), but no other statistically significant differences were observed.

FMI and %FM were associated with a worse performance in the 20 m shuttle run, standing long jump and 4 × 10 m shuttle run tests. More specifically, in adjusted analyses, each unit (kg/m^2^) increase in FMI was associated with a worse performance in the 20 m shuttle run (−0.50 laps, *p* = 0.002), standing long jump (−2.8 cm, *p* = 0.003) and 4 × 10 m shuttle run (+0.45 s, *p* < 0.001) tests. Similarly, %FM in adjusted analyses was associated with a worse performance for the 20 m shuttle run, standing long jump, and 4 × 10 m shuttle run tests (*p* ≤ 0.001). Further adjustment for VPA had very little influence on the observed associations between FMI and %FM and physical fitness.

FFMI was associated with all measures of physical fitness. In adjusted analyses, each unit (kg/m^2^) increase in the FFMI was associated with a better performance in the 20 m shuttle run (+0.48 laps, *p* = 0.002), handgrip strength (+0.64 kg, *p* < 0.001), standing long jump (+3.4 cm, *p* < 0.001) and 4 × 10 m shuttle run (−0.33 s, *p* = 0.004) tests. After adjusting these models for VPA, the observed associations between FFMI and physical fitness were slightly attenuated; however, they remained statistically significant (all *p* ≤ 0.043).

**Figure 1:**
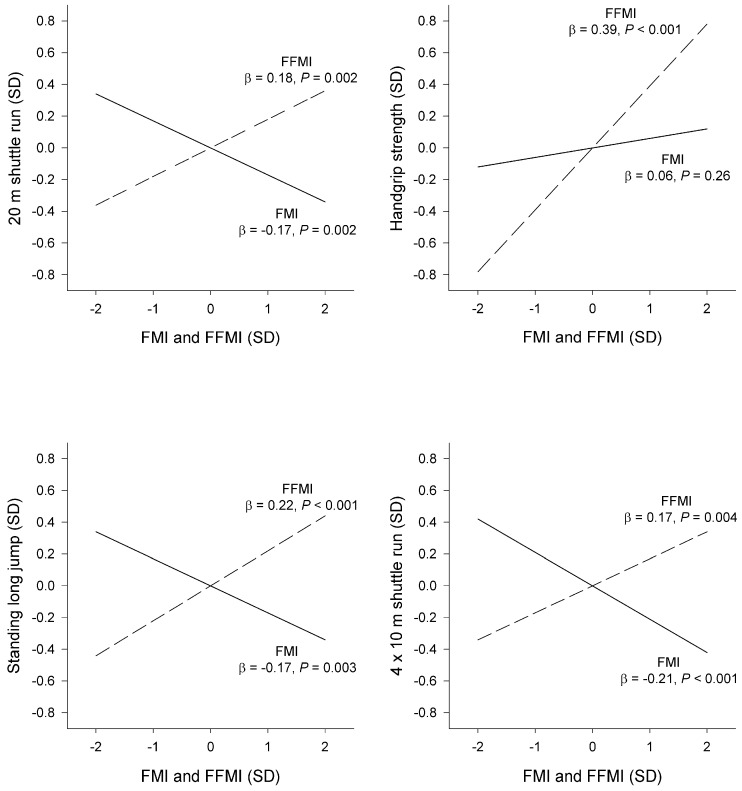
Graphical representation of the standardized regression coefficients (β) for the associations of fat mass index (FMI; solid lines) and fat-free mass index (FFMI; dashed lines) with measures of physical fitness. The standardized regression coefficients refer to the change in physical fitness (in SD) that are associated with a 1-SD increase in FMI and FFMI. Regression models were adjusted for age and sex and mutually adjusted for FMI and FFMI. Please note that the original 4 × 10 m shuttle run variable was inverted (− to + and vice versa) before being represented in this figure. Thus, this test can now be interpreted similar to the remainder of the physical fitness tests, i.e., a higher score indicates better performance.

We further explored associations of FMI and FFMI with physical fitness by visual representation of the standardized β-coefficients from the adjusted models, i.e., the change in physical fitness (in SD) that is associated with a 1-SD increase in FMI and FFMI (Figure 1). Thus, FMI (β = −0.17 to −0.21) and FFMI (β = 0.17–0.22) appear to have joint but opposite associations with physical fitness in the weight-bearing fitness test (i.e., shuttle runs and standing long jump). Please note that the slopes for the 4 × 10 m shuttle run were inverted since a lower score in this test (in seconds) indicates higher performance. Furthermore, FFMI had a strong influence (β = 0.39) on handgrip strength.

## 4. Discussion

### 4.1. Statement of Principal Findings

We observed that a higher FMI was associated with worse physical fitness in all weight-bearing tests (i.e., 20 m shuttle run, standing long jump and 4 × 10 m shuttle run tests), whereas a higher FFMI was associated with a better performance in all fitness tests in 4-year-old children. Hence, FM and FFM appear to have joint but opposite associations with physical fitness. Another interesting finding was that BMI was not associated with physical fitness in weight-bearing tests. Thus, the detailed and accurate body composition data in this study made it possible to identify associations between body composition and physical fitness. These findings expand the current literature since this is, to the best of our knowledge, the first study reporting associations of measures of both FM and FFM with physical fitness in preschoolers.

### 4.2. Comparison with Other Studies in Preschoolers

Reeves et al. [14] reported that a higher %FM, as measured by skinfolds, was associated with increased running times for the half mile run test in preschoolers. This finding can be compared to Agha-Alinejad et al. [15] who found that higher %FM as measured by skinfolds was associated with lower cardiorespiratory fitness in 5–6-year-old boys. Niederer et al. [13] assessed the physical fitness of 4–6 year olds and reported that overweight/obese preschoolers had lower cardiorespiratory fitness and agility compared to their normal-weight peers. Although Niederer et al. [13] did not report the association between %FM and physical fitness, they stated that their results would have been similar if classification was based on %FM using bioimpedance instead of BMI. We could confirm these findings using detailed body composition data since both FMI and %FM were associated with worse performance in the 20 m shuttle run (i.e., cardiorespiratory fitness) and the 4 × 10 m shuttle run (i.e., motor fitness) tests. Furthermore, we observed an association between higher FMI and %FM and worse performance in the standing long jump test which, to our knowledge, has not been reported in preschoolers previously. Our observed associations remained statistically significant after adjustment for objectively measured physical activity, which agree with the results reported by Niederer et al. [13]. No previous study has reported associations between FFM and physical fitness in preschoolers. Thus, our reported associations of a higher FFMI and a higher physical fitness represent novel findings.

Few studies have investigated the associations between BMI and physical fitness in preschoolers. Although not a universal finding [16], there is some evidence that a higher BMI is associated with lower cardiorespiratory fitness [11,12,13,14,15], motor fitness/agility [12,13,15] and lower-body muscular strength [17] suggesting that a higher FM is related to a lower performance in these fitness tests. Indeed, our results show that a higher FMI, and also a lower FFMI, is associated with a worse performance in the 20 m shuttle run (i.e., cardiorespiratory fitness), standing long jump (i.e., lower-body muscular strength) and 4 × 10 m shuttle run (i.e., motor fitness) tests. Furthermore, previous studies have reported that a higher BMI is associated with a higher handgrip strength in preschoolers [12,16]. Our results confirm that BMI is associated with handgrip strength and that the fat-free component of the weight, i.e., the FFMI, was positively related to handgrip strength. However, we observed no associations of BMI/BMI-groups with cardiorespiratory fitness, lower-body muscular strength and motor fitness in the current study. One reason for this discrepancy may be that the children in our study were as young as 4 years, because Niederer et al. [13] observed large differences in cardiorespiratory fitness and agility between normal-weight and overweight/obese 6-year olds, but interestingly not in 4-year olds.

### 4.3. Comparison with Studies in Older Children

Our results agree with previous findings in older children. In general, a higher FM or BMI has consistently been associated with a lower cardiorespiratory fitness in children [5,6,7,8,30] and adolescents [31,32]. With regards to muscle strength, studies have reported that a higher FFM or BMI is positively associated with handgrip strength [30,31,33], whereas a higher FM or BMI has been associated with worse performance in jumping tests [30,31,32]. These previous findings agree with our reported associations of FFMI or BMI with handgrip strength as well as between FMI and standing long jump. Evidence has also been presented indicating that a higher FM/BMI is related to worse performance in the 4 × 10 m shuttle run test [31,32] or 40 m test [30] (i.e., motor fitness), which agrees with our findings.

### 4.4. Direction of the Association between Body Composition and Physical Fitness

The direction of the cross-sectional associations between body composition and physical fitness is not obvious. Martinez-Tellez et al. [12] argued that health-related physical fitness can be seen as a marker of adiposity and consequently defined BMI as the independent variable. We therefore further investigated associations of physical fitness (x) with body composition (y) in Table S2. The results were, as expected, very similar to the ones presented in Table 2. Thus, a better performance in weight-bearing tests was associated with a lower FMI (all *p* ≤ 0.012) and a better performance in all tests was associated with a higher FFMI (all *p* ≤ 0.015). Hence, our results may also be interpreted such that higher physical fitness levels are associated with a more favorable body composition profile, i.e., less FM and/or more FFM, already in 4-year-old children.

We analyzed the associations of body composition (x) with physical fitness (y) since we deemed it more likely that body composition influences the physical fitness performance, than vice versa, in cross-sectional data. This is in accordance with most studies on preschoolers [11,13,14,16]. Nevertheless, further longitudinal studies on preschoolers are warranted to further examine the direction of the associations between body composition and physical fitness.

### 4.5. Strengths and Limitations

This study has several limitations. Due to the cross-sectional design of the study, causality and direction of associations cannot be concluded. Furthermore, the parents of the children in this study were well educated, which may limit generalizability. Nevertheless, the children in this study were very similar to average Swedish children with regards to weight and height as indicated by the z-scores in Table 1. The strengths of this study were that body composition was measured using accurate methodology [24] and the comprehensive physical fitness assessment was performed using the PREFIT battery [25], which has been evaluated in preschoolers [34]. Other strengths were that we could provide estimates of FMI and FFMI that were mutually adjusted for each other and the objective accelerometer data were included to adjust associations.

### 4.6. Interpretation and Implications

Zaqout et al. [30] observed that lower a BMI was associated with better performance in weight-bearing physical fitness tests and suggested that BMI represents a modifiable factor to improve physical fitness in children. Importantly, our results suggest that it is the components of BMI, i.e., less FMI and more FFMI, and not BMI per se that influences the physical fitness of children. The combined influence of FM and FFM on physical fitness in this study was relatively strong. More specifically, a decrease in the FMI and an increase in the FFMI corresponding to one SD was associated with a better performance in the 20 m shuttle run (+0.35 SD), standing long jump (+0.39 SD) and 4 × 10 m shuttle run (+0.38 SD) in adjusted models. Furthermore, a one SD increase in the FFMI was associated with a 0.39 SD higher hand grip strength. We therefore believe our identified associations of FM and FFM with physical fitness are strong enough to be relevant. These findings may be of public health importance since physical fitness already in childhood is related to future health [4,9]. However, as pointed out by Ortega et al. [25], there is a great need for future studies investigating the influence of physical fitness, in the preschool-age, on later health outcomes.

## 5. Conclusions

Our results demonstrate that a higher fat mass in 4-year-olds was associated with worse physical fitness in weight-bearing tests. The findings also clearly show that a higher fat-free mass was related to better levels of all physical fitness components. Thus, fat mass and fat-free mass appears to have joint but opposite associations with physical fitness, which is an important marker for current and future health.

## Figures and Tables

**Table 1:** Descriptive characteristics of the preschool children.

	All		Boys		Girls		*p* ^a^
	*n*	Value	*n*	Value	*n*	Value	
Age (years)	303	4.48 ± 0.15	168	4.49 ± 0.15	135	4.47 ± 0.15	0.34
Weight (kg)	303	18.3 ± 2.5	168	18.5 ± 2.4	135	18.1 ± 2.6	0.11
Weight for age *z*-score ^b^	303	−0.07 ± 1.11	168	−0.07 ± 1.11	135	−0.07 ± 1.10	0.97
Height (cm)	303	107.6 ± 4.2	168	107.9 ± 4.3	135	107.1 ± 4.1	0.10
Height for age *z*-score ^b^	303	−0.04 ± 0.97	168	−0.03 ± 1.00	135	−0.05 ± 0.94	0.85
Proportion of overweight/obesity ^c^	303	26 (8.6%)	168	14 (8.3%)	135	12 (8.9%)	0.86
BMI (kg/m^2^)	303	15.8 ± 1.4	168	15.8 ± 1.3	135	15.7 ± 1.4	0.28
FMI (kg/m^2^)	303	4.1 ± 0.9	168	4.0 ± 0.8	135	4.3 ± 1.0	0.001
FFMI (kg/m^2^)	303	11.6 ± 1.0	168	11.9 ± 1.0	135	11.4 ± 0.9	<0.001
FM (%)	303	26.0 ± 4.4	168	25.0 ± 3.9	135	27.3 ± 4.7	<0.001
Physical fitness test characteristics							
20 m shuttle run (laps)	296	5.9 ± 2.6	162	5.7 ± 2.6	134	6.1 ± 2.6	0.20
Handgrip strength (kg)	302	6.4 ± 1.6	168	6.8 ± 1.6	134	6.0 ± 1.4	<0.001
Standing long jump (cm)	303	71.7 ± 15.2	168	72.3 ± 15.6	134	70.9 ± 14.6	0.43
4 × 10 m shuttle run ^d^ (s)	303	18.2 ± 1.9	168	18.2 ± 2.2	134	18.0 ± 1.6	0.34
ActiGraph characteristics							
Valid days ^e^	295	6.7 ± 0.8	166	6.7 ± 0.7	129	6.6 ± 1.0	0.089
Awake wearing time (min/day)	295	841 ± 56	166	840 ± 59	129	843 ± 53	0.67
VPA ^f^ (min/day)	295	7.4 ± 4.9	166	8.1 ± 5.6	129	6.5 ± 3.6	0.004

Data are means ± standard deviation or *n* (%). BMI, body mass index; FM, fat mass; FFMI, fat-free mass index; FMI, fat-mass index; VPA, vigorous-intensity physical activity. ^a^ Refers to the *p* value of an independent test (continuous variables) or chi square test (categorical variables) between boys and girls; ^b^ Calculated using Swedish reference data [23]; ^c^ Classified according the World Obesity federation [22] cut-off; ^d^ In this test, lower scores (in seconds) indicate higher performance; ^e^ Defined as ≥600 min of nonsleeping data [27]; ^f^ Classified according to Chandler et al. [28].

**Table 2:** Associations of Body Composition with Physical Fitness ^a^.

				Body Composition Measures (x)								
	BMI (kg/m^2^)			FMI (kg/m^2^)			FFMI (kg/m^2^)			FM (%)		
**Physical fitness tests (y)**	b	SE	*p*	b	SE	*p*	b	SE	*p*	b	SE	*p*
20 m shuttle run (laps)												
Unadjusted	0.01	0.11	0.92	−0.46	0.16	0.006	0.44	0.16	0.006	−0.12	0.03	<0.001
Adjusted ^b^	0.01	0.11	0.95	−0.50	0.16	0.002	0.48	0.16	0.002	−0.12	0.03	<0.001
Adjusted ^b^ + VPA	−0.05	0.10	0.61	−0.45	0.15	0.003	0.33	0.15	0.030	−0.11	0.03	0.001
Handgrip strength (kg)												
Unadjusted	0.40	0.06	<0.001	0.07	0.10	0.50	0.72	0.09	<0.001	−0.04	0.02	0.070
Adjusted ^b^	0.38	0.06	<0.001	0.10	0.09	0.26	0.64	0.09	<0.001	−0.01	0.02	0.64
Adjusted ^b^ + VPA	0.37	0.06	<0.001	0.11	0.09	0.24	0.61	0.09	<0.001	−0.01	0.02	0.79
Standing long jump (cm)												
Unadjusted	0.43	0.64	0.50	−2.66	0.94	0.005	3.30	0.88	<0.001	−0.74	0.19	<0.001
Adjusted ^b^	0.36	0.64	0.57	−2.77	0.94	0.003	3.38	0.91	<0.001	−0.69	0.20	0.001
Adjusted ^b^ + VPA	0.09	0.64	0.88	−2.71	0.94	0.004	2.85	0.92	0.002	−0.65	0.20	0.001
4 × 10 m shuttle run ^c^ (s)												
Unadjusted	0.05	0.08	0.55	0.41	0.12	0.001	−0.28	0.11	0.015	0.10	0.03	<0.001
Adjusted ^b^	0.05	0.08	0.53	0.45	0.12	<0.001	−0.33	0.12	0.004	0.10	0.03	<0.001
Adjusted ^b^ + VPA	0.09	0.08	0.27	0.41	0.12	<0.001	−0.23	0.11	0.043	0.09	0.02	<0.001

BMI, body mass index; FM, fat mass; FFMI, fat-free mas index; FMI, fat-mass index; VPA, vigorous-intensity physical activity; x, independent variables; y, dependent variables. ^a^ Analysed using regression analysis. The unstandardized regression coefficient (**b**) with its standard error (SE) and the *p* value (*p*) are given for each association; ^b^ All models were adjusted for child´s sex and age. Models with FMI as the independent variable were further adjusted for FFMI while models with FFMI as the independent variable were further adjusted for FMI; ^c^ In this test, lower scores (in seconds) indicate higher performance. Hence, higher FFMI and lower FMI were associated with higher performance in the 4 × 10 m shuttle run test.

## Supplementary Materials

The following are available online at www.mdpi.com/2072-6643/8/8/473/s1, Table S1: Body composition and physical fitness in non-overweight and overweight preschool children, Table S2: Associations of physical fitness with body composition.

## Abbreviations

The following abbreviations are used in this manuscript:

BMI	body mass index
FFM	fat-free mass
FM	fat mass
FFMI	fat-free mass index
FMI	fat-mass index
VPA	vigorous-intensity physical activity
%FM	percent fat mass
