# Supplementary material for: Associations of Fat Mass and Fat-Free Mass with Physical Fitness in 4-Year-Old Children: Results from the MINISTOP Trial
Source: Nutrients. 2016 Jul 30;8(8):473. doi: 10.3390/nu8080473 (PMC4997386; doi:10.3390/nu8080473)
Supplement: Supplementary file 1 [file nutrients-08-00473-s001.docx]

**Supplementary Materials: Associations of Fat Mass and Fat-Free Mass with Physical Fitness in 4-Year-Old Children: Results from the MINISTOP Trial**

Pontus Henriksson, Cristina Cadenas-Sanchez, Marja H. Leppänen, Christine Delisle Nyström, Francisco B. Ortega, Jeremy Pomeroy, Jonatan R. Ruiz and Marie Löf

**Table S1.** Body composition and physical fitness in non-overweight and overweight preschool children.

|  | **Non-Overweight ^a,b^ (*n* = 277)** | **Overweight ^a,c^ (*n* = 26)** | ***p* ^d^** |
| --- | --- | --- | --- |
| Weight (kg) | 17.9 ± 1.9 | 23.0 ± 2.9 | <0.001 |
| FMI (kg/m^2^) | 4.00 ± 0.77 | 5.54 ± 1.17 | <0.001 |
| FFMI (kg/m^2^) | 11.51 ± 0.85 | 13.04 ± 1.02 | <0.001 |
| FM (%) | 25.7 ± 4.3 | 29.7 ± 4.4 | <0.001 |
| 20 meter shuttle run (laps) | 5.8 ± 2.6 | 6.0 ± 2.4 | 0.83 |
| Handgrip strength (kg) | 6.3 ± 1.6 | 7.5 ± 1.7 | <0.001 |
| Standing long jump (cm) | 71.5 ± 15.1 | 72.9 ± 16.2 | 0.67 |
| 4 × 10 meter shuttle run ^e^ (s) | 18.1 ± 1.9 | 18.6 ± 2.0 | 0.18 |

Data are means ± standard deviation. FM, fat mass; FFMI, fat-free mas index; FMI, fat-mass index.
^a^ Overweight (including obesity) was classified according to Cole and Lobstein (Pediatr Obes. 2012;**7**:284-94) and children not overweight (i.e., underweight and normal-weight) were classified as non-overweight; ^b^ 277 children (154 boys and 123 girls); ^c^ 26 children of which 14 were boys (12 overweight and 2 obese) and 12 were girls (10 overweight and 2 obese); ^d^ Refers to the *p* value of independent *t*-test between normal-weight and overweight children; ^e^ In this test, lower scores (in seconds) indicate higher performance.

**Table S2.** Associations of physical fitness with body composition ^a^.

|  | **Physical Fitness Tests (x)** | | | | | | | | | | | |
| --- | --- | --- | --- | --- | --- | --- | --- | --- | --- | --- | --- | --- |
|  | **20 m Shuttle Run (Laps)** | | | **Handgrip Strength (kg)** | | | **Standing Long Jump (cm)** | | | **4 × 10 m Shuttle Run ^b^ (s)** | | |
| **Body composition (y)** | b | β | *p* | b | β | *p* | b | β | *p* | b | β | *p* |
| BMI (kg/m^2^) |  |  |  |  |  |  |  |  |  |  |  |  |
| Unadjusted | 0.003 | 0.01 | 0.92 | 0.29 | 0.34 | <0.001 | 0.003 | 0.04 | 0.50 | 0.02 | 0.03 | 0.55 |
| Adjusted ^c^ | 0.002 | 0.00 | 0.95 | 0.30 | 0.35 | <0.001 | 0.003 | 0.03 | 0.57 | 0.03 | 0.04 | 0.53 |
| FMI (kg/m^2^) |  |  |  |  |  |  |  |  |  |  |  |  |
| Unadjusted | −0.056 | −0.16 | 0.006 | 0.02 | 0.04 | 0.50 | −0.010 | −0.16 | 0.005 | 0.09 | 0.14 | 0.001 |
| Adjusted ^c^ | −0.056 | −0.16 | 0.006 | 0.06 | 0.10 | 0.079 | −0.009 | −0.14 | 0.012 | 0.09 | 0.14 | 0.001 |
| FFMI (kg/m^2^) |  |  |  |  |  |  |  |  |  |  |  |  |
| Unadjusted | 0.059 | 0.16 | 0.006 | 0.27 | 0.44 | <0.001 | 0.013 | 0.21 | <0.001 | −0.07 | −0.14 | 0.015 |
| Adjusted ^c^ | 0.058 | 0.16 | 0.006 | 0.24 | 0.39 | <0.001 | 0.012 | 0.19 | <0.001 | −0.07 | −0.14 | 0.015 |
| FM (%) |  |  |  |  |  |  |  |  |  |  |  |  |
| Unadjusted | −0.34 | −0.20 | <0.001 | −0.29 | −0.10 | 0.070 | −0.063 | −0.22 | <0.001 | 0.52 | 0.23 | <0.001 |
| Adjusted ^c^ | −0.34 | −0.20 | <0.001 | −0.08 | −0.03 | 0.64 | −0.056 | −0.19 | 0.001 | 0.52 | 0.23 | <0.001 |

BMI, body mass index; FFMI, fat-free mas index; FM, fat mass; FMI, fat-mass index; x, independent variables; y, dependent variables. ^a^ Analysed using regression analysis. The unstandardized regression coefficient (**b**), standardized regression coefficient (β) and the *p* value (*p*) are given for each association; ^b^ In this test, lower scores (in seconds) indicate higher performance. Hence, a higher performance in the 4 × 10 m shuttle run test was associated with higher FFMI and lower FMI; ^c^ All models were adjusted for child’s sex and age.
